# Draft Genome Sequence of *Proteus mirabilis* NO-051/03, Representative of a Multidrug-Resistant Clone Spreading in Europe and Expressing the CMY-16 AmpC-Type β-Lactamase

**DOI:** 10.1128/genomeA.01702-15

**Published:** 2016-02-11

**Authors:** Marco Maria D’Andrea, Tommaso Giani, Lucia Henrici De Angelis, Nagaia Ciacci, Marek Gniadkowski, Vivi Miriagou, Francesca Torricelli, Gian Maria Rossolini

**Affiliations:** a Department of Medical Biotechnologies, University of Siena, Siena, Italy; b Department of Biology, University of Rome Tor Vergata, Rome, Italy; c Department of Molecular Microbiology, National Medicines Institute, Warsaw, Poland; d Laboratory of Bacteriology, Hellenic Pasteur Institute, Athens, Greece; e Genetic Diagnostic Unit, Florence Careggi University Hospital, Florence, Italy; f Department of Experimental and Clinical Medicine, University of Florence, Florence, Italy; g Clinical Microbiology and Virology Unit, Florence Careggi University Hospital, Florence, Italy

## Abstract

*Proteus mirabilis* NO-051/03, representative of a multidrug-resistant clone expressing the CMY-16 AmpC-type β-lactamase and circulating in Europe since 2003, was sequenced by a MiSeq platform using a paired-end approach. The genome was assembled in 100 scaffolds with a total length of 4,197,318 bp. Analysis of the draft genome sequence revealed the presence of several acquired resistance determinants to β-lactams, aminoglycosides, phenicols, tetracyclines, trimethoprim, and sulfonamides, of one plasmid replicon, and of a type I-E clustered regularly interspaced short palindromic repeat (CRISPR)-associated protein (Cas) adaptive immune system.

## GENOME ANNOUNCEMENT

*Proteus mirabilis* is one of the leading causes of urinary tract infections, especially in patients with functional or structural abnormalities of the urinary tract or with indwelling catheters, but it can also cause infections of other sites and bacteremia (1). In this species, which lacks functional chromosomal β-lactamase genes, acquired β-lactamase genes play an important role in the evolution of antibiotic resistance (2).

*P. mirabilis* NO-051/03 (also referred as IT NO-051/03) (3) was isolated in 2003 from a skin and soft tissue infection sample from a hospitalized patient from northern Italy (Novara) (4). The strain was resistant to penicillins and expanded-spectrum cephalosporins, due to the production of an acquired AmpC-type β-lactamase (ACBL) of the CMY lineage, named CMY-16 (3–5), and also to fluoroquinolones. The strain, which caused a clonal outbreak in northern Italy, was subsequently found to be highly related to a clone spreading in Europe and North Africa since the 1990s (3).

Bacterial DNA was subjected to whole-genome sequencing with a MiSeq platform (Illumina, Inc., San Diego, CA), using a 2 × 250-bp paired-end approach. In total, 2,216,404 reads were obtained and assembled using ABySS (6) into 100 scaffolds (>208 bp in size), with a total length of 4,197,318 bp and an *N*_50_ of 160,469 bp. The genome raw coverage was ≈130×. The average G+C content was 39%.

A total of 3,778 coding sequences (CDSs), 82 tRNAs, 48 rRNAs, and 2 clustered regularly interspaced short palindromic repeat (CRISPR) systems were annotated by PGAP (http://www.ncbi.nlm.nih.gov/genome/annotation_prok). Further analysis showed the presence of a type I-E CRISPR-associated protein (Cas) adaptive immune system characterized by 20 spacers, of which 16 lacked any detectable identity with sequences in nucleotide databases, while 3 matched prophage sequences, and one matched a DNA fragment present between the *mucB* and *resD* genes in several conjugative IncL/M-type plasmids. These data suggest the ability of NO-051/03 to resist invasion by a number of phages and IncL/M-type plasmids.

The antimicrobial resistome of NO-051/03 was investigated with ResFinder (7), which confirmed the presence of two acquired β-lactamase genes (*bla*_CMY-16_ and *bla*_TEM-1b_) and detected additional acquired resistance genes to aminoglycosides (*strAB*, *aacA4*, *aac(*3*)*-*I*, *aadA1*, and *aph(3′)-Ic*), aminoglycosides and quinolones (*aac(6′)Ib-cr*), chloramphenicol (*cat* and *catA1*), tetracyclines (*tet*(A)), trimethoprim (*dfrA1*), and sulfonamides (*sul1* and *sul2*). Further analysis of the quinolone resistance-determining regions of topoisomerase genes (8) showed the presence of mutations previously linked to reduced susceptibility to fluoroquinolones, namely, those encoding S83R in GyrA, S464Y in GyrB, and S80R in ParC.

PlasmidFinder (9) detected the presence of an IncQ1 replicon, while PHAST (10) revealed the presence of 5 intact, 2 incomplete, and 2 putative prophages.

To the best of our knowledge, this is the first genome sequencing project of a *P. mirabilis* isolate producing an ACBL. The results from this project are expected to broaden the knowledge on the genetic factors that have contributed to the successful international spread of such isolates*.*

### Nucleotide sequence accession numbers.

The complete genome sequence of *P. mirabilis* NO-051/03 was deposited at DDBJ/EMBL/GenBank databases under the accession number LGAY00000000. The version described in this paper is the first version, LGAY01000000.

## References

[1] Flores-Mireles AL, Walker JN, Caparon M, Hultgren SJ 2015 Urinary tract infections: epidemiology, mechanisms of infection and treatment options. Nat Rev Microbiol 13:269–284. doi:10.1038/nrmicro3432. 25853778 PMC4457377

[2] Livermore DM 1995 beta-Lactamases in laboratory and clinical resistance. Clin Microbiol Rev 8:557–584. 8665470 10.1128/cmr.8.4.557 PMC172876

[3] D’Andrea MM, Literacka E, Zioga A, Giani T, Baraniak A, Fiett J, Sadowy E, Tassios PT, Rossolini GM, Gniadkowski M, Miriagou V 2011 Evolution and spread of a multidrug-resistant *Proteus mirabilis* clone with chromosomal AmpC-type cephalosporinases in Europe. Antimicrob Agents Chemother 55:2735–2742. doi:10.1128/AAC.01736-10. 21402851 PMC3101460

[4] D’Andrea MM, Nucleo E, Luzzaro F, Giani T, Migliavacca R, Vailati F, Kroumova V, Pagani L, Rossolini GM 2006 CMY-16, a novel acquired AmpC-type beta-lactamase of the CMY/LAT lineage in multifocal monophyletic isolates of *Proteus mirabilis* from northern Italy. Antimicrob Agents Chemother 50:618–624. doi:10.1128/AAC.50.2.618-624.2006. 16436718 PMC1366893

[5] Luzzaro F, Brigante G, D’Andrea MM, Pini B, Giani T, Mantengoli E, Rossolini GM, Toniolo A 2009 Spread of multidrug-resistant *Proteus mirabilis* isolates producing an AmpC-type beta-lactamase: epidemiology and clinical management. Int J Antimicrob Agents 33:328–333. doi:10.1016/j.ijantimicag.2008.09.007. 19095415

[6] Simpson JT, Wong K, Jackman SD, Schein JE, Jones SJ, Birol I 2009 ABySS: a parallel assembler for short read sequence data. Genome Res 19:1117–1123. doi:10.1101/gr.089532.108. 19251739 PMC2694472

[7] Zankari E, Hasman H, Cosentino S, Vestergaard M, Rasmussen S, Lund O, Aarestrup FM, Larsen MV 2012 Identification of acquired antimicrobial resistance genes. J Antimicrob Chemother 67:2640–2644. doi:10.1093/jac/dks261. 22782487 PMC3468078

[8] Weigel LM, Anderson GJ, Tenover FC 2002 DNA gyrase and topoisomerase IV mutations associated with fluoroquinolone resistance in *Proteus mirabilis*. Antimicrob Agents Chemother 46:2582–2587. doi:10.1128/AAC.46.8.2582-2587.2002. 12121936 PMC127365

[9] Carattoli A, Zankari E, García-Fernández A, Voldby Larsen M, Lund O, Villa L, Møller Aarestrup F, Hasman H 2014 *In silico* detection and typing of plasmids using PlasmidFinder and plasmid multilocus sequence typing. Antimicrob Agents Chemother 58:3895–3903. doi:10.1128/AAC.02412-14. 24777092 PMC4068535

[10] Zhou Y, Liang Y, Lynch KH, Dennis JJ, Wishart DS 2011 PHAST: a fast phage search tool. Nucleic Acids Res 39:W347–W352. doi:10.1093/nar/gkr485. 21672955 PMC3125810

